# Co-option of an endogenous retrovirus (LTR7-HERVH) in early human embryogenesis: becoming useful and going unnoticed

**DOI:** 10.1186/s13100-025-00361-0

**Published:** 2025-07-05

**Authors:** Zsuzsanna Izsvák, Jin Ma, Manvendra Singh, Laurence D. Hurst

**Affiliations:** 1 The Max Delbrück Center for Molecular Medicine in the Helmholtz Association (MDC), Robert-Rössle-Straße 10, Berlin, 13125 Germany; 2 Department of Molecular Biology & Genetics, Cornell University, 526 Campus Road, Ithaca, NY 14853 USA; 3 The Milner Centre for Evolution, Department of Life Sciences, University of Bath, Bath, BA2 7AY UK

## Abstract

While it is straightforward to understand why most mutations affecting functional sequence are harmful, how genomic changes result in new beneficial traits is harder to understand. Domestication of transposable elements (TEs) is an important source of both new genes and new regulatory systems as, for their own propagation, TEs need to have transcription factor binding sites and functional products that predispose to their recruitment. But are such predispositions to gain-of-function sufficient? Here we consider the case of the endogenous retrovirus, HERVH. Knockdown data supports HERVH having roles in pluripotency, self-renewal and defence against transpositionally-active retroelements in the early human embryo. We clarify the pluripotent cell types associated with HERVH expression and, in the process, note a key unresolved issue, framed by the unwanted transcript hypothesis: how can some cell types have 2% of their transcripts being HERVH-derived but survive the multiplicity of cellular devices that suppress foreign transcripts, be this by transcriptional repression or post-transcriptional filtering? We note a common coupling between novelty generation and suppression evasion. For example, pluripotency-associated KLF4 binding is thought to compete with transcriptional suppressor binding. Similarly, HERVH has a strong splice site enabling efficient novel chimeric transcript formation, the resulting exon–intron junctions enabling evasion of the unwanted transcript filters that recognize low or absent intron presence. We conclude that to better understand domestication, a focus on predispositions to avoidance of unwanted transcripts filters, as well as predispositions to gain of functions, is necessary. The same insights will be valuable for transgene design (eg for gene therapy) and instructive of gain-of-function in tumours, as HERVH is known to be involved in onco-exaptation events.

**Supplementary Information:**

The online version contains supplementary material available at 10.1186/s13100-025-00361-0.

As organisms are well adapted beings, it is straightforward to see why most mutations affecting functional sequence—be they point mutations, deletions or insertions—would be deleterious and in turn why most selection is purifying. This is evidenced, for example, by the observation that the rate of non-synonymous evolution in coding sequence is typically much lower than at synonymous sites (i.e. Ka/Ks < < 1) [1], even in mammals, this notwithstanding the fact that purifying selection is expected to be inefficient when the effective population size is low [2] (e.g. in mammals). That selection would favour some novel functions is also clear, but mechanistically how – mutationally speaking – novel functions are generated is not so transparent. This is somewhat akin to asking how one might take a functioning watch and improve it by tinkering with, or adding, components. Why many random changes would degrade, or indeed, break, the watch is transparent (many might also be effectively neutral). The nature of the changes that add to the watch’s functionality are not so clear.

Here we do not intend to review this literature (for recent considerations see [3, 4]). Rather, we note that transposable elements (TEs) are common contributors to gain of functions. Often this is attributed to the fact that to be a successful genomic invader TEs need to have transcription factor binding sites and functional transcriptional and protein products that predispose to their recruitment via domestication [5]. A paradigmatic example is the case of *Syncytins*: retroviral envelope (*env*) genes, originally responsible for membrane fusion, were independently recruited twice in mammals to facilitate membrane fusion in the cells of the trophoblast during placental development [6]. Interestingly, another *env*-derived gene, *Suppressyn*, encoded by HERVH48, retains the receptor-binding domain of the *env* protein but lacks fusogenic function [6]. Instead, it acts as a negative regulator of syncytia formation mediated by syncytin-1 [7], and has been proposed to contribute to antiviral defense mechanisms [8]. We can also point to LTRs of endogenous retroviruses as having multiple transcription factor (TF) binding sites, a prerequisite for being a successful invader but also predisposing to gain, or transfer, of functionality [5, 9, 10].

 While such functional predispositions are likely to be important, are they sufficient to understand how functions are gained? Here we consider the case of endogenous retroviruses (ERVs), in particular HERVH this being recruited to pluripotency, self-renewal and anti-mobile element defence in the early human embryo. We argue, in the context of the unwanted transcript hypothesis [11] that predisposition to functionality alone is not sufficient. Rather, there exists a multiplicity of cellular devices to suppress, at all stages of gene expression, the expression of unwanted transcripts, transcripts that serve no utility for the host be these actively parasitic, remnants of parasitic elements, spurious transcripts, mis-spliced forms etc. Indeed, we suggest that the fact that as many as 2% of all transcripts in some early embryo cells are HERVH-derived is the most remarkable of observations: very recently, in evolutionary time, similar HERVH transcripts should have been recognised as foreign and suppressed. How then can they be so abundant, and functional and survive anti-foreign transcript systems? Evasion of the multiple devices to counter unwanted transcripts is, we suggest, key to TE domestication. Just as certain TEs may have predispositions to enable functionality for the host, so any predisposition to escape suppression will make those sequences more likely to be domesticated, although mutations gained later on, possibly as an adaptive response to suppression, may also be important. While, in retrospect, this is possibly an obvious point, more striking is the sometimes intimate coupling between novelty generation and escape from suppression, as we here discuss.

We start by laying out the biology of ERVs, consider LTR7/HERVH as an example of co-option and outline the evidence for its functionality. We take this opportunity to clarify the HERVH-related activity in different classes of pluripotent cells. We then highlight unknowns, a key one of which is how HERVH transcripts can be so very common in some of these cell types. Examining in detail how suppression is avoided we note that HERVH has some unusual features that simultaneously promote novelty and should act to avoid the filters that evolved to prevent expression. Understanding how some transcripts avoid suppressive filters is, we suggest, not only relevant to the broad question of how over the short evolutionary time novelty evolves. These lessons may well help to understand why some transgenes come to nothing, while others are robustly expressed. This should be of utility for designing transgenes for stable integration, eg for gene therapy. In addition, understanding why HERVH is involved in gain-of-functions on a tumour, rather than evolutionary, timeline (eg onco-exaptations) may well cast light on the same issues.

## Endogenous retrovirus as cellular invaders

Endogenous Retroviruses (ERVs) are ancient retroviral integrations that have become part of the host genome. Initially, ERVs retain the ability to exit the host cell, but over time, they lose this capacity and begin to follow the evolutionary life cycle of transposable elements (TEs) [12]. The endogenization process requires integration into the germline, but similar to other TEs, ERVs can also transpose into other lineages [12–14]. This dynamic process allows active TEs to move in and out of the germline, potentially influenced by environmental conditions [15, 16]. In mammals there is a specific connection between the germline and pluripotency as development involves the transition from pluripotency to epiblast after implantation, from which the germline develops so that primordial germ cells (PGCs) regain pluripotency [17]. Indeed, PGCs express transcription factors that are also expressed in pluripotent stem cells (PSCs) and characterise the pluripotent state [18]. This provides important insights on how TEs are co-opted for the fitness of germ cells so that they are transmitted to the next generations and ultimately fixed in populations.

**Fig. 1:**
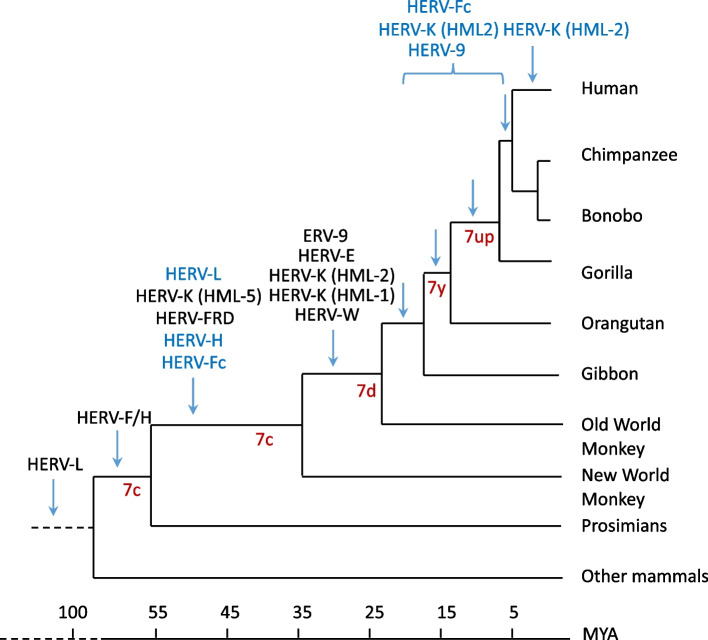
Invasion of the ancestor of various HERV families during primate evolution. Note that HERVs (in blue) invaded multiple times (adopted from ref [29]). Evolution of HERVH subfamilies (in dark red) during primate evolution adopted from ref [31]

While TEs are active, they can be under selection to have a reduced degree of harm and so self-suppress to some degree [19, 20]. Nonetheless, any activity is likely to incur costs to the hosts and hence there is a continuous "arms race" between them and their host. Throughout evolution, hosts have developed a variety of mechanisms to suppress active TEs, while TEs have concurrently evolved strategies to evade these defenses (reviewed in refs [15, 21]). Despite these ongoing interactions, the typical evolutionary outcome for TE/ERVs is eventual inactivation. Ectopic recombination is one of the inactivation processes that frequently affect ERVs due to sequence identity in their long terminal repeats (LTRs), leading to deletion of most of the ERV sequence and leaving only ‘*solo*’ LTRs [22, 23]. After inactivation, accumulation by drift of mutations renders the sequences unrecognisable as TE-derived over time. (H)ERVs make up about 8% of the human genome [24, 25]. The annotated HERVs are categorised into class I (gamma-like), class ii (beta-like) and class III (spuma-like) (Table 1). Among the oldest HERVs, the HERVL elements are estimated to have integrated into the genome over 100 million years ago (MYA), predating the divergence of placental mammals [26], whereas HERVK are the most recent [27] (Fig. 1), still capable of producing viral particles [28].

**Table 1:** Copy numbers of different HERVs in the human genome, flanked by LTRs or present as *solo* elements. The table was adapted from ref [29]. Repbase name of the long terminal repeat (LTR) follows in parentheses. Most copy numbers are from ref [30]. Additional information was reported on HERVH [31–34] and HERVK [35]. Note that the ratio of copies flanked by LTRs and *solo* elements are the highest in HERVH (in bold)

Class	HERV family	Repbase	Copies	Solo	Ratio Copies/S olo
Class I	ERV-3	HERV3(LTR4)	100	125	0,8
	ERV-9	HERV9 (LTR12,PTR5)	300	5000	0,06
	HERV-ADP	HERVP-71A I (LTR71A)	40	300	0,13
	HERV-E	HERVE (LTR2,2B,2C)	250	1000	0,25
	HERV-F	HERVFH19I (LTR19)	45	550	0,08
	HERV-Fb	HERVH48I (MER48)	60	100	0,06
	HERV-Fc	HERV46I (LTR46)	2	4	0,5
	HERV-FRD	MER50I (MER50)	50	2000	0,025
	**HERV-H**	**HERVH (LTR7)**	**1000–1200**	**1000, 1270**	**0,8-1**
	HERV-HS49C23	MER57I (MER57)	200	1000	0,2
	HERV-I	HERVI (LTR10)	250	1000	0,25
	RRHERV-I	HERV15I (LTR15)	40	250	0,16
	HERV-P	HUERS-P3 (LTR9)	200	1000	0,2
	HERV-Rb	PABL BI (PABL A/B)	8	1000	0,008
	HERV-T	HERVS71 (LTR6A,B)	80	400	0,2
	HERV-W	HERV17 (LTR17)	40	1100	0,36
	HERV-FXA	HERVFH21 (LTR21A)	30	40	0,75
Class II	HERV-K(HML-1)	HERVK14I (LTR14A,B)	68	350	0,19
	HERV-K(HML-2)	HERVK (LTR5)	60, 91	2500, 944	0,024–0.097
	HERV-K(HML-3)	HERVK9I (MER9)	150	700	0,214
	HERV-K(HML-4)	HERVK13I (LTR13)	10	800	0,0125
	HERV-K(HML-5)	HERVK22I (LTR22,-A,-B)	100	600	0,17
	HERV-K(HML-6)	HERVK3I (LTR3, 3B)	50	400	0,125
	HERV-K(HML-7)	HERVK11D1 (MER11D)	20	140	0,14
	HERV-K(HML-8)	HERVK11I (MER11A,B,C)	60	600	0,1
	HERV-K(HML-9)		10	40	0,25
	HERV-K(C4)	HERVKC4 (LTR14)	10	100	0,1
	HERV-K(14C)	HERVK14CI (LTR14C)	15	120	0,125
Class III	HERV16	HERV16 (LTR16, 16A-D)	15	25	0,6
	HERV-L	HERVL (MLT2A1–2B2)	580	6000	0,97
	HERV-S	HERV18 (LTR18, 18B)	50	150	0,33

However, not all TEs become mere genomic fossils; some undergo a rare process of exaptation (alias co-option) (Table 2). This process can potentially begin while the TE or ERV is still active, providing certain advantages to the host, which may lead to its tolerance. Due to their functional and structural similarities, interactions between exogenous viruses and specific ERVs have been observed. For example, HIV-1 or Epstein-Barr virus (EBV) infection can lead to the upregulation of certain ERV elements, which may affect the host's immune response [36–38], but can also interfere with the production of HIV-1 viral particles [39]. Additionally, inactive ERV-derived sequences, such as certain open reading frames (ORFs) (eg *env*, *gag*), transcription regulatory sequences, and transcription factor (TF) binding sites, can be repurposed to support host functioning [23, 40, 41].

**Table 2:** The best-characterized co-opted HERVs in the human genome [42]

HERV family	Co-opted function	Role in humans	HERV-derived product
HERV-W (ERVW-1)	Placental trophoblast fusion	Promotes syncytiotrophoblast formation for fetalmaternal exchange	*env*-derived protein Syncytin-1
HERV-FRD	Immunosuppression in pregnancy	Helps to prevent the maternal immune system from attacking the semi-allogeneic fetus	*env*-derived protein Syncytin-2
HERVH48	Antiviral defence	Negative regulator of trophoblast differentiation	*env*-derived protein Suppressyn
HERVL	Marking 8-cells tage^a^	By analogy to MERVL (mice), it might regulate embryonic gene activation (EGA) in early embryogenesis	HERVL-driven gene regulation chimeric transcripts^a^
HERVK (HML-2)	Antiviral defence Regulatory activity in 8/16 cell to early blastocyst stage stage^a^	Protection against de novo retroviral infection Unclear	*env*-derived protein (*Rec*) Potential enhancer HERVK-driven transcripts^a^
LTR7-HERVH	Antiviral/TE defence Regulatory activity in the human inner cell mass (ICM- EPI) stages	Self-renewal and pluripotency of stem cells	*gag*-derived sequences HERVH-derived transcripts lncRNA Chimeric transcripts TADs miRNA sponge

^a^needs further clarification

### LTR7-HERVH as an example of ERV co-option

RTVL- retrovirus, the ancestor of HERVH invaded the ancestor of the primate genome around 40 MYA [25, 43]. The invasion occurred multiple times (Fig. 1), and during the endogenization process, which began around 35 MYA [43], the number of HERVH elements expanded through both reinfection and retrotransposition within the genome. Recombination occurred between the different subfamilies [31]. By analysing genomic structures of HERVH elements, it was observed that in contrast to conventional ERVs, the HERVH elements are more frequently flanked by two LTRs than expected from their evolutionary age [43, 44]. The ratio of close-to full length versus *solo* copies is exceptionally (1:1) high, still *solo* LTRs still constitute a significant proportion of HERVH elements in the human genome (Table 1). Despite the fact that HERVH is no longer transposing, approximately 1000–1200 HERVH copies are flanked by LTRs [31–34, 45]. This observation led to the hypothesis that these (or at least some of these) HERVH copies likely have co-opted function(s).

The evolution of the LTR7 sequence (Fig. 1) was shaped by a combination of mutational processes, including point mutations, duplications, and multiple recombination events between subfamilies, which resulted in transcription factor binding motif modules unique to each subfamily [31]. Currently, four subfamilies of HERVH elements are listed in the Dfam [46] and Repbase [47, 48] databases, and they are annotated in the reference human genome based on distinct LTR consensus sequences: LTR7 (formerly Type I), 7b (Type II), 7c, and 7y (Type Ia) [47, 49]. Additional subdivisions of HERVH elements have been proposed based on phylogenetic analyses and structural variations in their internal gene sequences [23, 32, 50] as well as their LTRs [31]. The different subfamilies are expressed in well-defined developmental niches [31, 51]. Curiously, the subfamilies (eg LTR7up1/2) expressed in pluripotent stem cells exhibit a robust increase in transcription [31].

If they are domesticated, what might their functions be? One possible clue comes from their time of activity. Currently, around 300 genomic loci of full-length LTR7-HERVH are expressed in human pluripotent stem cells. One of the most highly transcribed loci is the HERVH-derived novel gene, ESRG [52].

Over the past decade, research has gradually uncovered several co-opted functions of HERVH (Table 2), demonstrating its multifaceted contributions to genomic regulation and cellular processes (reviewed in ref [53]). The investigation into HERVH's co-opted functions began with the observation that HERVH has binding sites for multiple key pluripotency factors (POU5F1/OCT4, NANOG, TFCP2L1/LBP9, SOX2, and KLF4) [51, 52, 54, 55]. That this binding is functionally relevant is evidenced by the observation that depletion of HERVH RNA in human pluripotent stem cells (hPSCs) leads to a loss of self-renewal and upregulation of differentiation markers [52, 55, 56]. Since then, HERVH expression has been recognized as a marker for pluripotent stem cells, as its activation promotes both the acquisition and maintenance of pluripotent states [57–60]. Notably, LTR7-HERVH-derived transcripts are detected in the human embryo, and embryos are not viable in its absence [61], suggesting their co-option in early human embryogenesis.

The modes of activity of HERVH appears to be highly diverse, and not as simple as old TE creates a de novo protein (i.e. not like syncytins). HERVH loci may function as robust alternative promoters and enhancers [9, 51, 62, 63], with several copies overlapping with annotated super-enhancers [64]. Activated HERVH loci frequently generate long non-coding RNAs (lncRNAs) or produce chimeric transcripts with neighbouring protein-coding genes [52, 65]. These transcripts, which contain HERVH-derived and unique chimeric sequences, play specific roles in hPSCs, such as ESRG [52], lncROR [66], and Lnc00458 [67]. There are approximately 50 HERVH genomic loci that form topologically associating domain (TAD) boundaries in hPSCs, especially the highly transcribed ones [68], which actively shape cell type-specific chromatin architecture. Highly transcribed HERVH genomic loci can also establish topologically associating domain (TAD) boundaries in hPSCs [68], actively shaping cell type-specific chromatin architecture. Additionally, HERVH-derived lncRNAs contribute to chromatin remodeling and gene activation during differentiation processes [67, 69]. The relative functional relevance of each of these activities is unknown.

**Fig. 2:**
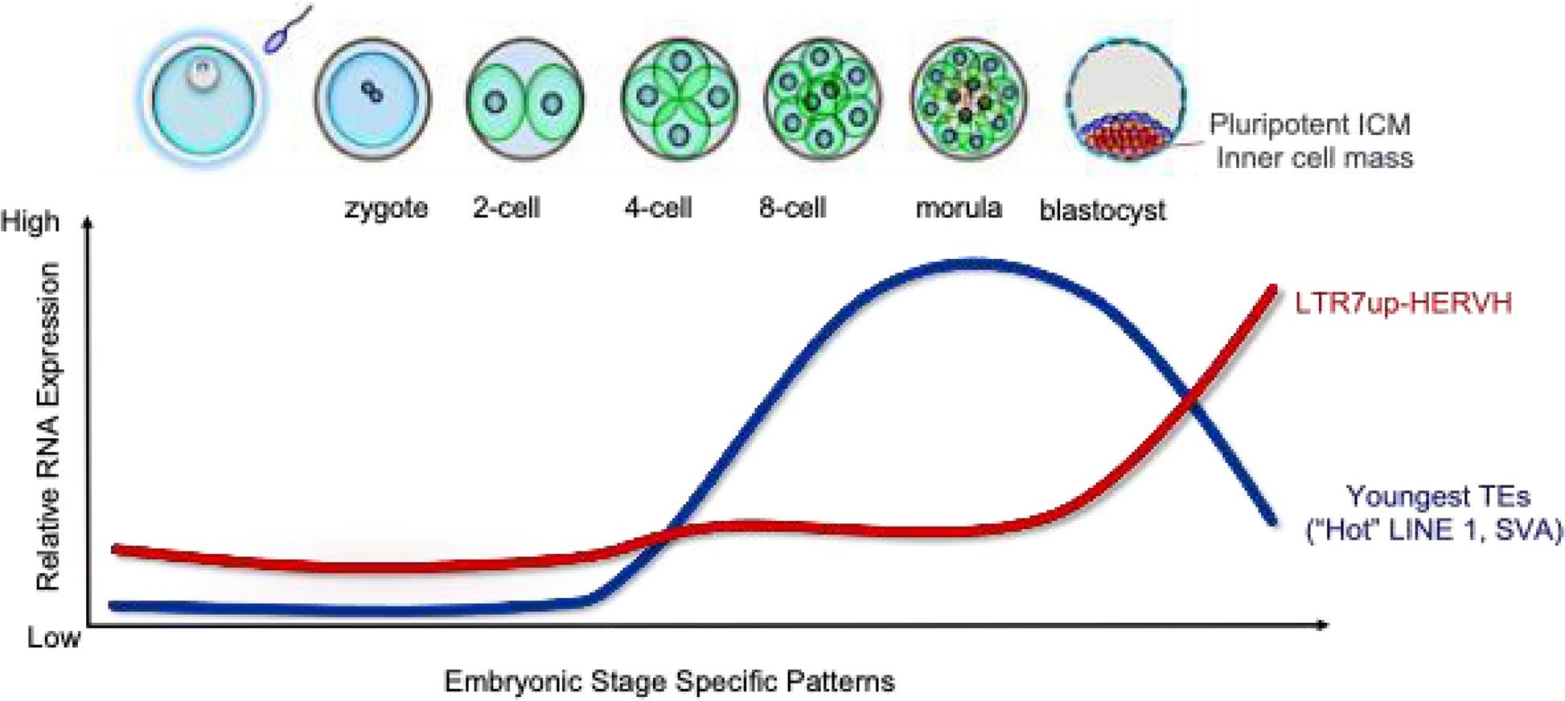
Antagonistic expression pattern of phylogenetically Young (< 7MY old, eg “Hot” Line 1, SVA) and Old (> 7 MY old, eg HERVH) elements during human early embryonic development (data source: GEO-GSE 36552)

Beyond supporting self-renewal and pluripotency, LTR7-HERVH products are also involved in host defence, such as conserved *gag* sequences that may act as antagonists against viral reinfection [23] or by suppressing the activity of other TEs and ERVs [70]. Consistent with such a role, there is antagonistic expression pattern between potentially mutagenic retrotransposable elements (REs) and LTR7-HERVH around the fifth day of embryonic development (after the morula stage) (Fig. 2). HERVH knockdown is indeed associated with depression of younger REs [70]. Mechanistically there may be several modalities to any suppression. HERVH acts as a cis-regulatory element upstream of APOBEC3G as an enhancer for several APOBEC3 genes [63]. The APOBEC3 genes (apolipoprotein-B mRNA-editing catalytic polypeptide-like-3) are an essential component of the innate immune response in humans and play a crucial role in limiting the activity of retroviruses (eg HIV-1) and the autonomous retroelement LINE-1 [71–73]. APOBEC3G particularly targets single-stranded DNA and induces G-to-A hypermutations in retroviral DNA. The HERVH-APOBEC3 locus may also be part of a broader HERVH-based defense mechanism against potentially harmful genomic invaders in early embryogenesis. Analysis of alternative modalities of young RE suppression by HERVH is an active area of research.

There may be other utilities of HERVH. In addition to generating lncRNAs and chimeric transcripts, full-length HERVH copies occasionally encode open reading frames (ORFs), such as *gag* [23]. However, unlike the more evolutionarily recent HERVK, which is shown to produce viral particles [28], HERVH does not seem to do so. Instead, the preservation of the domain containing zinc finger motifs within the HERVH *gag* ORF suggests a co-opted function [23]. It has been proposed that the original function of these zinc finger motifs—enabling retroviral proteins to package RNA for the benefit of an exogenous retrovirus—has been repurposed to bind viral RNA, potentially providing an immunological benefit to human cells [23]. This repurposing of viral genes to function as host immunity genes is commonly referred to as endogenous viral element-derived immunity (EDI) [74].

Notably, HERVH elements flanked by LTRs were identified, rather than *solo* LTR sequences in co-opted roles, although the *solo* LTRs may still act as enhancers and contribute to tissue-specific or developmental gene expression patterns, which needs to be validated.

### HERVH expression in human pluripotent* in vitro *stem cell cultures

While HERVH has been proposed as a precise marker of human pluripotency [57], it is worthwhile expanding on this as there are at least three distinct pluripotent stem cell types that exhibit different but characteristic expression profiles of HERVH and other transposable elements (TEs).

#### Primed pluripotent stem cells

The primed state of human ESC pluripotency resembles that of mouse epiblast stem cells (EpiSCs) more closely than that of mouse ESCs [75]. In primed pluripotent stem cells, HERVH genomic loci are actively transcribed (*n* = 250–300) (Table 3), and these loci largely overlap with those expressed in various induced pluripotent stem cell lines (hiPSCs) [52].

**Table 3:** Features of three human pluripotent cell types. Characteristically expressed TE families are as in [52, 76]

Feature	Primed	Naïve	eFORM
Developmental stage	Post-implantation epiblast	Pre-implantation epiblast/ late morulaearly blastocyst	Pre-implantation epiblast/ blastocyst
Pluripotency	Primed for differentiation	Ground state	Ground state
Ability to revert to primed pluripotency	N/A	Low	High
Morphology	Flattened colonies, 2D	Round colonies, 3D	Round colonies, 3D
Epigenetics	Genomic hypermethylation, Xa/Xi inactivation	Genomic hypomethylation, Xa/Xa	Genomic hypomethylation, Xa/Xa
Proliferation rate	24–36 h	18–24 h	42–48 h
Characteristically expressed TE families	L1_Hs L1_PA2	L1_Hs SVA_D LTR5-HERVK LTR7Y_B-HERVH	LTR7-HERVH
Expressed HERVH loci	250–300	50–80	350–400
Genome stability	Stable	Less stable, frequent chromosomal abnormalities	Stable

#### Naïve pluripotent stem cells

When primed hESCs/hiPSCs are cultured in naïve media [76, 77], the cells transition to a so-called naïve stage. Although mouse and human naïve pluripotent stem cells share a similar morphology (eg, form dome-shaped colonies) and certain well-defined characteristics, the naïve stage is not identical in mice and humans. In mice, naïve cells model the inner cell mass (ICM) of the blastocyst, while in humans, they are thought to represent a developmental stage prior to ICM formation, closely resembling the morula/early blastocyst stage [76]. In terms of TE profiling, human naïve cells exhibit relatively low expression of HERVH (~ 50–80 genomic loci) [76] (Table 3). Conversely, these cells display high expression of phylogenetically younger TEs (< 7MY), such as active L1_Hs and SVA [76]. It is noteworthy that younger L1 elements are partly responsible for determining the pluripotent stage of the mouse [78], whereas the human pluripotent stage has an opposite dynamic, as it has apparently developed through the suppression of younger elements.

#### eFORM (Naïve-like) human pluripotent stem cells

This cell type, which constitutes approximately 4% of human pluripotent stem cell cultures (hESC/hiPSCs), is morphologically similar to human naïve pluripotent stem cells [52]. However, unlike naïve cells, this "naïve-like" cell type is characterized by high and stable expression of LTR7-HERVH (HERVH^High^) derived from around 350–400 genomic loci (Table 3) and negligible expression of younger REs (eg. LINE-1, SVA, HERVK) [63]. HERVH expression also provides telomere protection [52]. Given that LINE-1 expressing cells segregate from the forming embryo while LTR7-HERVH-derived transcripts contribute to human embryogenesis [61], we have named this HERVH^High^ cell type as naïve-like embryo-forming (eFORM).

HERVH^High^ eFORM cells retain their ability to self-renew and can be maintained long-term [59]. However, the elevated levels of HERVH inhibit differentiation [58, 79]. For a successful transition out of the self-renewing pluripotent state, HERVH expression must be reduced. This downregulation is partially mediated by the BTB domain-containing zinc finger protein ZBTB12 [80] or by TUT7 [81].

### HERVH transcript abundance and other enigmas in early development

Despite significant progress in understanding the impact of LTR7-HERVH on human physiology, some unresolved issues remain. One concerns the different functionalities – if any – of the different LTRs. Typically activated from the eight-cell stage, HERVH can be driven by variants of LTR7 (eg B/C/Y), each providing transcription at slightly different pre-implantation developmental stages [31, 51]. These different LTR variants are thought to represent an ongoing arms race [82] between host defense mechanisms and HERVH. It is noteworthy that in addition to LTR7, the subfamily LTR7Y also has a 1:1 ratio between LTR-flanked and *solo* elements, whereas the *solo* LTRs in the subfamilies LTR7B and LTR7C exceed the number of nearly proviral copies [31]. Nevertheless, the question remains as to which subfamily, such as LTR7, was used for functional tasks or continues to be captured by host defence mechanisms.

Beyond LTR7B/Y, HERVK, and SVA (SINE-VNTR-Alu) elements are abundantly expressed from the eight-cell stage to the early blastocyst [76] (Fig. 2), suggesting potential functional co-option in early development (Table 2). Indeed, it is tempting to speculate that HERVK derived protein product (eg *Rec*) may serve a protective role against viral infection [28]. However, it remains puzzling that HERVK and SVA are co-expressed with LINE-1, a mutagenic retrotransposon, despite the fact that highly damaged cells are eliminated from the developmental program [63]. This raises the question: if LINE-1 compromises genome integrity, why are HERVK and SVA tolerated during the same developmental window? Given that host defense mechanisms are not yet fully functional at this stage, it remains an open question whether HERVK and SVA elements are indispensable for early development or if their expression is tolerated due to a permissive chromatin state**.**

An intriguing possibility is that HERVH serves as a regulatory hub, counteracting SVA/HERVK/LINE-1 expression to modulate transposon activity. This implies that HERVH may regulate pluripotency while simultaneously suppressing other transposable elements, acting as an early defense mechanism. Its inverse correlation with SVA/HERVK expression (Fig. 2) further supports this hypothesis, although this concept remains to be experimentally validated.

Despite multiple reports highlighting the significance of HERVH in pluripotent stem cells, a major concern remains the conflicting evidence regarding the role of the HERVH-derived ESRG locus in maintaining pluripotency. ESRG (Embryonic Stem Cell Related), is among the most abundantly transcribed LTR7-HERVH locus [52]. It contains at least one putative open reading frame (ORF) (Q1W209), but it remains unclear whether it is functional. Notably, the ORF is predicted to encode a 222-amino-acid protein unique to humans [52], though this prediction is primarily supported by transcription data. Furthermore, Wang et al. demonstrated that knocking down ESRG expression is leading to compromised self-renewal and pluripotency in hESCs [52]. However, this finding was challenged by Takahashi et al., who found ESRG to be dispensable in hPSC lines where it was specifically knocked out (KO) [83]. Li et al. [84] later provided a potential resolution to these conflicting results by suggesting that the temporal knockdown of TP53 during the construction of ESRG knockout cell lines [83] might have contributed to the discrepancies. The cumulative effect of this knockdown, particularly in a TP53-deficient background, could account for the divergent outcomes observed across studies. Additionally, knockout experiments using the CRISPR/Cas9 system may introduce off-target effects. Moreover, while the knockdown affects multiple HERVH copies, the knockout approach targets only a single genomic locus. If HERVH transcripts function within a network, maintaining a specific threshold of HERVH expression may be crucial for their biological role.

Following on from the high abundance of ESRG transcripts, more generally the relative abundance of HERVH transcripts in self-renewing pluripotent stem cells that will contribute to the embryo is noteworthy and enigmatic. Remarkably, the expression of HERVH-associated lncRNAs is highly efficient, with levels up to eightfold higher compared to non-HERVH-associated lncRNAs in hPSCs [54]. Intriguingly, HERVH products account for approximately 2% of all transcripts in the nuclei of these self-renewing pluripotent stem cells [52, 55, 57]. What remains unclear is first, why are they so very common and second, why HERVH-derived transcripts, unlike younger REs, are not recognized as foreign by the host defense system during early embryogenesis. In the very recent evolutionary past, ERVH would have been recognised as a dangerous endogenous retrovirus. Nonetheless, despite their high expression levels, these transcripts do not trigger an immune response, which suggests that at least certain HERVH copies may have evolved mechanisms to evade detection, or that the early embryonic environment has unique features that allow tolerance of these elements. Understanding this selective recognition—or lack thereof—by the host defense system could provide insights into the regulation of genomic elements during development.

### Distinguishing self from non-self, and the “Unwanted Transcript Hypothesis”

The problem of recognition of transcripts derived from HERVH sits within a broader context of control and filters on transcriptions of all types. Current estimates suggest that over 80% of our genome is transcribed [85, 86]. Given that approximately half of the human non-coding genome originates from invasive elements, such as previous viral infections and TE activities, this results in a significant amount of transcriptional junk. The challenge for organisms with large amounts of invasive elements is managing the vast number of transcripts produced by the non-coding genome. According to the ‘unwanted transcript hypothesis’ [11], cells have evolved sophisticated mechanisms to suppress ‘unwanted’ transcripts or, if produced, filter them out and silence ‘non-self’ elements while maintaining the expression of essential host genes. Transcriptional suppression of transcripts recognized as foreign by the HUSH complex, nuclear degradation by the nuclear exosome complex and control of nuclear export are three of the suggested mechanisms [11]. Beyond transcriptional control, transcripts derived from TEs are indeed filtered out by several specialized mechanisms [87–103] and reviewed in refs [42, 104–109] (Table 4). These protective mechanisms are specialized, targeting different types of TEs/ERVs at various stages and levels, often complementing each other (for a review, see [42, 110, 111]).

**Table 4:** Host-encoded suppression mechanisms and their effects on TEs [42] [87–93, 95–106, 109], (r) review article. The symbols indicate references to topics listed in the “Key Players” column

Mechanism	Key Players	Mechanism of Action	Targeted TEs	Examples
Epigenetic Silencing	DNA Methylation* Histone Modification^#^ (H3K9me3, H3K27me3, H4K20me3), HUSH^@^ complex, SALl4^&^, CAF-1^	Compact chromatin to repress TE transcription	HERVs, LINE-1, SINEs, Satellite DNA	* [42] (r) # [87, 106] *(r); * [103] @ [88] [109](r) & not yet reported ^ [89]
Post-transcriptional silencing (RNAi)	piRNAs*, siRNAs#, miRNAs^@^, Argonaute, Dicer	Small RNAs guide silencing complexes to degrade TE transcripts and recruit repressive chromatin marks	ERVs, LINE-1, Alu	[105] (r) # [90] @ [91]
RNA Surveillance	RNA Exosome* NEXT^#^, PAXT^@^ Nonsense- Mediated Decay (NMD)^&^ StaufenMediated Decay (SMD)^	Decay pathways recognize and degrade TE-derived RNAs	TE-derived transcripts, long exon- containing RNA, (> 1 kb) intronretained RNAs, LINE-1, HERVK	* [92] # [93, 94] @ not yet reported & [104] (r) ^ [95]
Nuclear export	TREX/NXT* CRM!1 (Exportin-1)#,	Preventing cytoplasmic transfer and translation of TEs	HERVK (*Rec*), LINE-1,	* [96] ^#^ [97]
Sequestration	SAMHD1* NEAT1^#^	cytoplasmic stress granules nuclear paraspeckles	LINE-1 Alu	* [98] ^#^ [99]
Innate Immune Response	RIG-I*, MDA5^#^, ADAR^&^	Detect TE-derived dsRNA or cDNA, triggering an antivirallike response	Cytosolic LINE-1 DNA, dsRNA,	* [100] ^#^ [101] ^&^ [102]

**Fig. 3:**
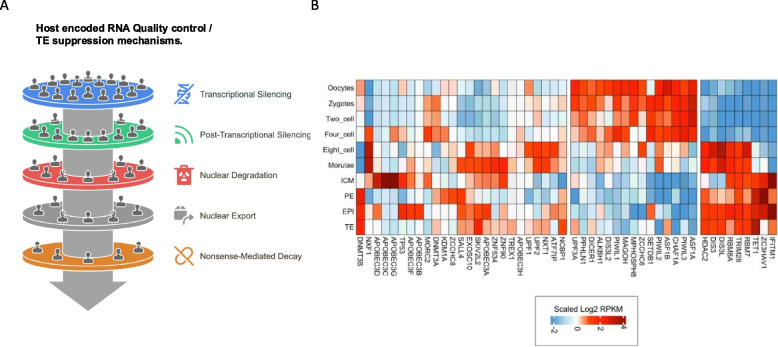
Host-encoded factors involved in RNA quality control (QC) and TE suppression mechanisms in early human embryonic development. **A** The schematics illustrates the QC-based filtering mechanism of unwanted (TE-derived) transcripts [11]. Transcriptional silencing (i) HUSH: Targets TEs, silencing through H3K9me3 deposition; (ii) SALL4: contributes to H3K27me3 deposition; (iii) CHAF1A: helps to promote the formation of a H3K9me3-marked heterochromatin; TP53: repression of TEs that contain P53 response elements; Post-transcriptional silencing PIWI-piRNA pathway; Nuclear degradation; Exosome targeting (NEXT/PAXT); Nuclear export, TREX/NXT; Nonsense mediated decay (NDM); slow translation; APOBEC3, promotes deaminase induced mutations; Innate immunity, IFIT1M; **B** Heatmap showing the differential expression of host-encoded factors involved in RNA quality control and TE suppression mechanisms in early human embryonic development (data source from [112])

In humans, transpositionally competent REs, such as LINE-1 and SVA get activated after embryonic gene activation (EGA). The developmental window that enables the activation of mutagenic REs (Fig. 2) suggests that the host's filtering and defence mechanisms are not yet fully functional (Fig. 3). We have provided evidence that these transposition-competent REs can indeed retrotranspose, leading to the elimination of severely damaged cells from the developmental program [63]. This previously unnoticed cell type, termed "REject," lacks commitment markers, expresses DNA damage response genes, is excluded from the developmental process, and eventually undergoes apoptosis [63].

Interestingly, the transcriptional decline of the mutagenic REs around day six of embryonic development is followed by the emergence of transcripts from hundreds of HERVH loci driven by LTR7, peaking at the 6-7th day of blastocyst stage (Fig. 2), such that LTR7(up)-HERVH activation appears to counteract the transcriptional upregulation of REs. In contrast to REject cells, which are eliminated from the forming embryo, HERVH-derived transcripts have been shown to contribute to the formation of the inner cell mass (ICM) of the human embryo [61, 63]. Since certain host defense mechanisms become active in the pluripotent epiblast (Fig. 3), it raises the question of why LTR7-HERVH products are not recognized as foreign and can bypass the host's defense system undetected. For example, interferon-induced transmembrane protein 1 (IFITM1), which has been reported to suppress the expression of certain HERVs (eg HERVK, LTR7Y-HERVH and LTR12D-1), is abundantly expressed in pluripotent stem cells (Fig. 3). However, it may selectively target LTR7Y-HERVH rather than LTR7-HERVH [113]. Even more convincing is the fact that LTR7up-HERVH together with IFITM1 increases the expression of APOBEC3G, APOBEC3C and [32], enzymes known to catalyse the conversion of cytosine to uracil in single-stranded DNA intermediates of various viruses and retroelements [114] (Fig. 3). This suggests that LTR7-HERVH may even play a role in the regulation of other REs.

### Becoming useful and going unnoticed

The notion that cells have elaborate systems of transcriptional suppression and of transcript filtering adds to the complexity of the domestication process. It isn’t good enough to observe that an HERVHs LTR has transcription factor binding sites, which certainly may predispose to domestication, but we need also to consider how a domesticated transcript escapes all the snares put in place. Here, then we discuss selected examples of HERVH co-option, highlighting how HERVH has evaded host defense mechanisms, been tolerated, and subsequently co-opted to support pluripotent stem cells in humans. The collected evidence suggests that HERVH has escaped suppression on multiple levels and even that the co-option and avoidance of suppression process may be mechanistically tightly coupled.

#### The pioneer transcription factor KLF4 may prevent TRIM28/KAP1 binding and recruit additional pluripotency factors

In pluripotent stem cells (PSCs), many ERV/TEs are silenced through tri-methylation of histone H3 at lysine 9 (H3K9me3), a process facilitated by the enzyme SetDB1 (SET domain bifurcated histone lysine methyltransferase 1). SetDB1 is recruited to ERV/TEs by sequence-specific DNA-binding proteins known as Krab zinc-finger proteins (KZFPs). These KZFPs interact with Trim28 (tripartite motif-containing 28, also known as KRAB-associated protein 1 or Kap1), along with other associated factors, to contribute to heterochromatin formation and DNA methylation. The Trim28 suppression machinery is particularly well-characterized in mice, where it plays a major role in regulating TE expression in mouse embryos [115–117].

**Fig. 4:**
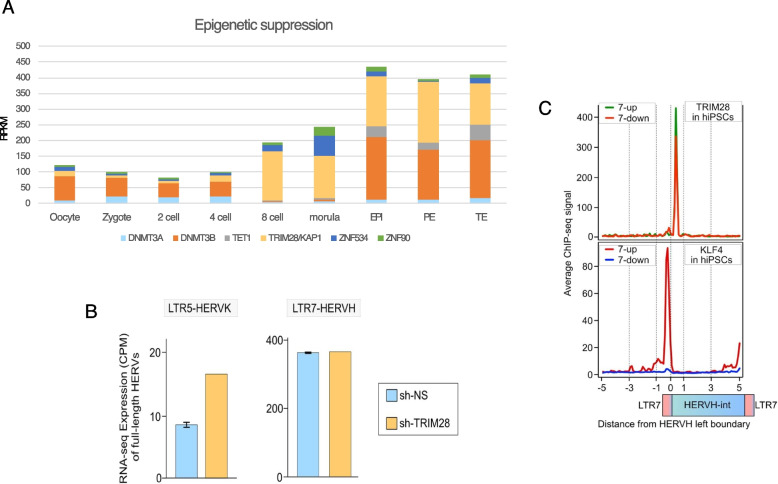
TRIM28 is not a significant regulator of LTR7/HERVH in human embryonic stem cells. **A** Expression of selected proteins involved in the epigenetic regulation REs, including the TRIM28 suppression system, the LTR7 specific ZNF90 and the LTR7up specific ZNF534 [31, 120] during human early embryonic development (data source from [112]). **B** LTR7-HERVH remains active under normal conditions, whereas LTR5-HERVK is derepressed upon TRIM28/KAP1 depletion (data source form [118]. **C** TRIM28 and KLF4 ChiP-seq counts over LTR7/HERVH genomic loci in hiPSC (data source from [58, 118]

In humans, TRIM28 is expressed as early as the 8-cell stage (Figs. 3 and 4A). In human pluripotent stem cells (hPSCs), TRIM28-mediated repression has been reported to broadly regulate a wide range of retroelements (REs) [118]. However, only a small subset of HERVH sequences (~ 0.06%) are directly bound by TRIM28 [76]. Notably, in TRIM28 knockout hPSCs [76, 119], the differentially expressed HERVH loci are predominantly associated with various LTR7 family members (eg LTR7B, LTR7C, LTR7Y) (Table 5 and Table S1). Furthermore, ESRG expression remains unaffected in TRIM28 knockout ESCs, and only three of the differentially expressed loci rank among the top 100 expressed HERVH loci (Table 5 and Table S1).

**Table 5:** Differentially expressed (DE) genomic loci of the LTR7 subfamily members in TRIM28 knockout (T28KO) hESCs [76]. Number of loci expressed in the 100 highest/lowest category. Number of LTR7s expressed in hESCs: 879 [119]

LTR7 subfamily	DE in T28KO	DE in highest 100	DE in lowest 100
**LTR7**			
Up	8	1	1
Down	1		
**LTR7B**		1	
Up	27		4
Down	0		
**LTR7C**			
Up	6		1
Down	0		
**LTR7Y**			
Up	2		
Down	1	1	
**Sum**	45	3	6

Beyond regulating TEs, TRIM28 may also play additional roles, such as controlling transcriptional elongation [121], which could explain its crucial function in ESCs. Notably, TRIM28 null mutations impair hPGCLC differentiation, indicating that its silencing in human PSCs compromises germline competence [119]. This underscores the essential role of TRIM28-mediated RE suppression in safeguarding the human germline.

Additionally, in our analysis of human embryonic stem cells depleted of TRIM28 using shRNA (shTRIM28-hESC_H1) [122], we observed no significant change in the number of expressed LTR7-HERVH genomic loci. While silencing of TRIM28 in human PSCs has no effect on the expression of HERVH, it leads to upregulation of other REs, including LTR5-HERVK (Fig. 4B) and SVA elements [118]. Together, these findings suggest that, in human PSCs, LTR7-HERVH largely escapes suppression by the TRIM28/KAP1 complex, unlike LTR5-HERVK.

Interestingly, de-repression of Young REs has little immediate effect and is even compatible with short-term self-renewal [80, 119]. In contrast, the biological response in mouse pluripotent cells is more pronounced, as a Trim28 null mutation in mouse cells is incompatible with embryonic stem cell (ESC) self-renewal [123], indicating species-specific differences in TRIM28 function during development. Beyond regulating REs, TRIM28 may also play additional roles, such as controling transcriptional elongation [121], which could explain its crucial function in ESCs. It is notable that TRIM28 null mutations are detrimental to hPGCLC differentiation, suggesting that silencing TRIM28 in human PSCs impairs germline competence [119]. This finding highlights the critical role of TRIM28-mediated RE suppression in protecting the human germline.

**Fig. 5:**
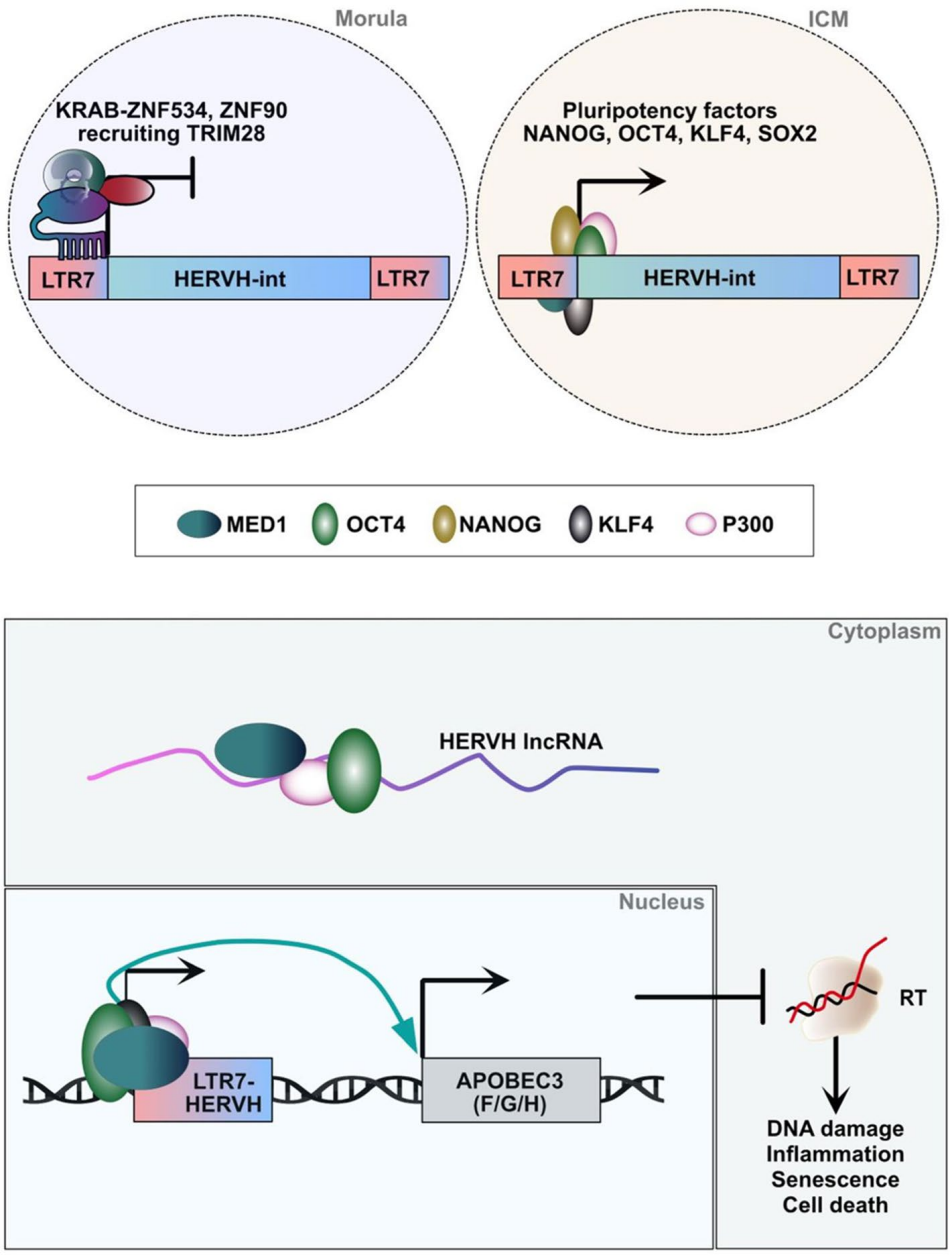
(Upper panel) The KRAB-ZNF/TRIM28 machinery is a suppressor of the LTR7-HERVH loci in the morula, but not in the ICM, where its expression is controlled by pluripotent transcription factors. (Middle panel) HERVH lncRNA recruits OCT4, P300 and MED1 in the cytoplasm and (Lower panel) enables expression of the LTR7-HERVH/APOBEC3 locus in the nucleus. APOBEC3F/G/H inhibit reverse transcription (RT) activity

Why, then, does TRIM28 repress many retroelements (REs) but largely not HERVH in pluripotent stem cells? One possible explanation is that, although HERVH is specifically targeted by ZNF proteins (eg ZNF90, which recognizes all LTR7 subfamilies, and ZNF534, which preferentially binds LTR7up, these potential repressor proteins are highly expressed during the morula stage but are expressed at low levels in pluripotent stem cells [31] (Figs. 3 and 5). As a result, they suppress LTR7-HERVH only in cellular contexts where these factors are sufficiently expressed. A second explanation, supported by our TRIM28 ChIP-seq data analysis, is that TRIM28 binding does not lead to HERVH silencing per se. Notably, TRIM28 binds within the internal HERVH sequences and not in the regulatory LTR7 regions occupied by pluripotency factors (Fig. 4C). Alternatively, it is possible that TRIM28, when bound to HERVH, undergoes post-translational modifications that alter its function, potentially shifting its role from a repressor to an activator.

Furthermore, LTR7-HERVH provides binding sites for pluripotency transcription factors, including POU5F1/OCT4, NANOG, TFCP2L1/LBP9 and KLF4 [51, 52, 54, 55]. Given that TRIM28/KAP1 does not seem to be the primary suppressor of HERVH in human ESCs, it has been proposed that pioneer factor KLF4 [124] prevents TRIM28/KAP1 binding and instead recruit additional pluripotency factors, as well as the E1A-binding protein p300 histone acetyltransferase, to HERVH loci [56, 58]. In this model, both the novel pluripotency functions and escape from suppression are possible as KLF4 mediates both (Fig. 5).

In mouse pluripotent stem cells (PSCs), Trim28-induced suppression leads to DNA methylation, resulting in permanent silencing of TEs [125, 126], whereas the relationship between TRIM28 occupancy and DNA methylation is less tight in human PSCs [118]. Either way, the hypothesis that KLF4 outcompeting TRIM28 contributes to the evasion of suppressive methylation is worthy of further scrutiny. While DNA CpG methylation generally suppresses TEs, hypomethylation within specific TE families is associated with tissue-specific enhancer landscapes. Indeed, LTR7-HERVH is significantly hypomethylated in the genomes of PSCs [53, 127].

#### Underrepresented for canonical TP53 binding sites for transcriptional control

The tumor protein 53 (p53 alias TP53) plays a central role in regulating transposable elements in the germline through conserved mechanisms (reviewed in ref [128]). In germline, p53 enforces genome integrity by inducing cell death via a conserved apoptotic mechanism in cells with DNA damage or abnormal genomes [129, 130]. In human development, the movement of the only autonomous TE, LINE-1 (L1), was long thought to be restricted to the germline. However, we have recently reported that cells exhibiting DNA damage associated with L1 activity are also removed through apoptosis during early human embryogenesis, specifically after the morula stage [63]. Under physiological conditions, L1 activity has also been observed in human neuronal cells [131].

Besides its interplay with piRNA pathways, TP53 can directly suppress TE expression through DNA binding. TP53 binds conserved recognition motifs within LINE-1 elements, specifically at the 5' untranslated region (5’-UTR), where it represses L1 transcription by recruiting repressive chromatin modifiers, leading to heterochromatin formation [132, 133]. Interestingly, approximately 30% of TP53 binding sites overlap with genomic loci of endogenous retroviruses (ERVs) across the genome [134]. Some of these sequences were identified as negative regulators of ERV transcription (eg HERV‐1‐LTRs) [135]. Curiously, several (1,509/319,000) of the TP53 binding sites identified in ERVs are canonical (RRRCWWGYYY- 0-13 bp spacer- RRRCWWGYYY) [136]. Notably, these canonical TP53 sites are significantly underrepresented in LTR7-HERVH (Table S2). While this could mean that these HERVH loci have escaped TP53-mediated suppression, alternatively, the lack of TP53 binding sites may simply reflect either the absence of evolutionary selection for TP53 regulation or selection against it in pluripotent stem cells, where TP53 suppression of HERVH may not have provided a selective advantage.

Interestingly, TP53 binding sites do not always suppress L1 transcription. In response to double-strand breaks (DSBs), certain L1 loci increase transcription and transposition, creating a positive feedback loop that amplifies genomic stress and triggers TP53-mediated apoptosis [137]. Here, TP53 binds a slightly altered motif (p53 responsive element) within the L1 promoter, promoting L1 mRNA synthesis and retrotransposition. This paradoxical mechanism reinforces genomic stability by amplifying TP53-dependent responses that enforce fidelity through cell death.

During evolution, some TEs have acquired TP53 DNA binding sites, enabling them to regulate neighboring gene expression and form part of a broader regulatory network [132]. Intriguingly, non-canonical TP53 sites (differing in sequence and featuring a 129-bp spacer) have been identified in the *pol* domain of certain HERVH loci [137]. The biological significance of these non-canonical TP53 binding sites in HERVH warrants further investigation.

#### Releasing of LTR7/HERVH from transcriptional suppression

RNA N6-methyladenosine (m6A) modification is the most abundant epitranscriptomic modification [138]. Post-transcriptional modification of RNA by m6A plays a critical role in regulating the silencing of TEs by influencing histone modifications [139]. m6A-modified RNA are recognized by reader proteins, such as YTHDC1 and YTHDC2. When YTHDC1 binds to m6A-marked TE-derived transcripts, it can lead to either the direct degradation of these transcripts or the recruitment of histone modifiers, which induce the epigenetic silencing of genomic TE loci through histone modifications [140, 141].

HERVH appears to employ a similar suite of players but their effect is the reverse, i.e. activating of HERVH. The RNA m6A reader YTHDC2 specifically interacts with m6A-modified HERVH RNA transcribed from the LTR7-HERVH genomic loci in human PSCs. YTHDC2 not only occupies the genomic loci of LTR7-HERVH but also collaborates with the DNA 5mC ‘eraser’ TET1 demethylase. The removal of 5mC from LTR7-HERVH by TET1 helps maintain the active state of the LTR7-HERVH loci in hECSs, and influences their neuronal fate commitment [142]. This example illustrates how, in the context of a domestication process, instead of suppressing the LTR7-HERVH locus, the m6A-modified HERVH RNA recruit TET1, thereby releasing LTR7-HERVH from transcriptional suppression. It is as though HERVH has somehow hijacked the system sent to suppress it to activate instead.

#### Developmentally programmed HERVH degradation

Uridylation by TUT4/7 inhibits the retrotransposition of LINE-1 elements [143]. TUT7 also uridylates HERVH RNAs, leading to their degradation [81]. Interestingly, the regulation of HERVH through TUT7-mediated degradation is an integral part of the human developmental program, particularly in neural differentiation. Cells that maintain high levels of HERVH expression (HERVH^High^ cells) are deficient in neural differentiation [58, 79]. However, the programmed degradation of HERVH by TUT7 restores pluripotency and enables these cells to differentiate into the neural lineage [81].

#### HERVH in chimeric transcripts – splicing

In retroviruses, *pol* and *gag* are not processed by splicing, whereas splicing is essential for *env* expression. In HERVH, while *gag* remains recognizable [23], *pol* does not, and the HERVH loci lack an intact *env* gene due to mutations and deletions. Unlike HERVK, which retains protein-coding potential, HERVH is primarily transcriptionally active.

Splicing likely contributes to the stabilization of HERVH-derived long non-coding RNA (lncRNA) transcripts. Notably, compared to mRNAs, lncRNAs often undergo inefficient splicing, frequently utilizing cryptic splice sites or non-canonical mechanisms. Indeed, certain HERVH-derived lncRNAs, such as LINC-ROR, exhibit features of cryptic splicing or non-canonical mechanisms, such as intron retention and multiple isoforms [144]. Many of these variants are likely to be lost through random evolutionary processes unless maintained by positive selection. However, specific spliced isoforms that confer functional advantages could be retained.

Importantly, a subset of HERVH possesses functional splice donor (SD) and splice acceptor (SA) sites, enabling the formation of chimeric transcripts. This suggests that HERVH’s retroviral-derived splicing machinery has been repurposed to incorporate exons from human genes into novel chimeric transcripts. These spliced transcripts typically integrate HERVH as the 5’ sequence, bridging to the 5’ splice site of exon 2 of a neighboring gene on the same strand. In contrast, SAFB suppresses exonization of other retroelement-derived transcripts [145]. HERVH elements are also characterised by their ability to efficiently donate transcription start sites (TSSs) to chimeric transcripts [53]. These properties, together with their strong LTR, facilitate the formation of chimeric transcripts.

In addition to HERVH, SVA elements are also capable of generating chimeric transcripts. However, while some SVA elements have their own promoter [146], many are dependent on upstream gene promoters or read-through transcription from host genes. It has been shown that SVA elements are incorporated into newly formed transcripts as exonic sequences via their cryptic splice acceptor sites [147].

**Fig. 6:**
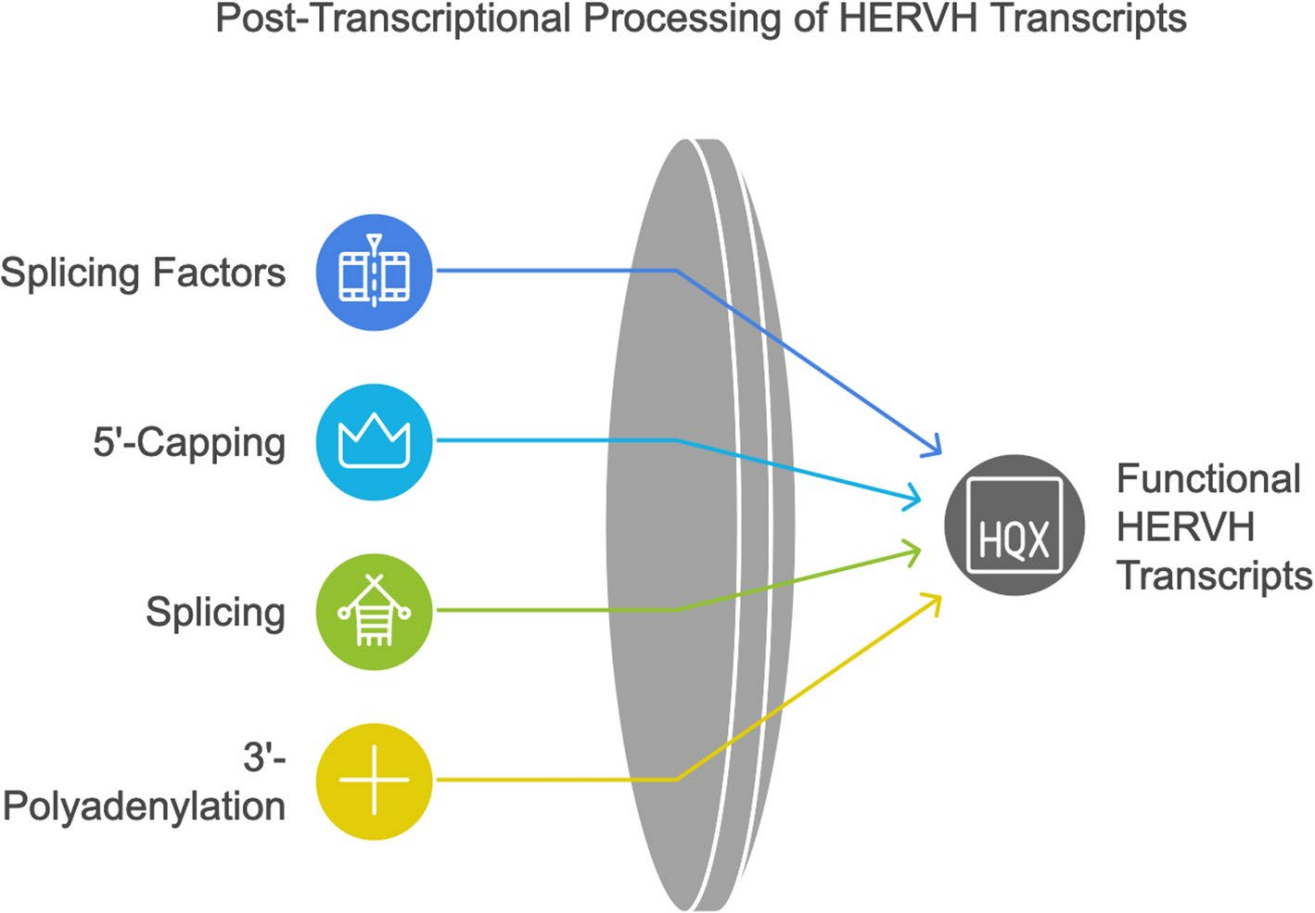
HERVH-derived transcripts effectively recruit splicing factors, likely undergo typical post-transcriptional processing steps, including 5′-capping, splicing and 3′-polyadenylation

At least a portion of the HERVH-derived chimeric transcripts with protein-coding genes are expected to be translated. HERVH-derived chimeric lncRNAs [52, 55], actively recruit splicing factors (eg SF1, SF3A1/B3) [84] and SFRS3/9/10/12 [61], suggesting that they undergo typical post-transcriptional processing, including 5′-capping, splicing, and 3′-polyadenylation (Fig. 6). The absence of splicing, as well as the positioning of the first intron near the ATG codon, plays a crucial role in distinguishing native transcripts from aberrant ones. Splicing is anticipated to enhance the stability and functionality of HERVH-mediated chimeric transcripts, in part by passing the first intron and multi-exon tests necessary for nuclear export. The first intron can also facilitate progressive RNA polymerase II elongation [148]. Additionally, a multi-short exon structure enhances transcript recognition [11].

These HERVH-enhanced chimeric products contribute to gene regulation within the pluripotent niche [52, 56].

## Discussion

How transposable elements are domesticated is of focal concern to the problem of the evolutionary gain of function problem (how to improve a swiss watch). While the focus has historically been on TE features recruited to host utility, there exists a second component, we suggest, that has attracted less attention, this being how a domesticated TE might avoid the filters and traps set up to prevent or remove unwanted transcripts. Our central hypothesis proposes that the co-option of HERVH has involved evasion from cellular surveillance mechanisms [11].

A subtle aspect of our model is that it requires that the anti-TE filters are leaky: if a TE escapes one suppressor it still needs to pass through the others which it cannot do if all filters are foolproof: escape from one is of no utility if it faces full suppression later. We argue that the evolution of anti-TE systems predisposes to a system with multiple leaky filters. First, any suppressive system needs to balance the need to effectively express native genes with filters to prevent the expression of non-native ones. It is hard to envisage any system that is so effective that it permits expression of all native genes and only these genes. A foolproof filter against intronless transcripts, for example, would capture many essential transcripts (eg histones) and likely be catastrophic. The filters may thus need to be leaky. Moreover, as with the case of mutation rate reduction, there comes a point where the benefits of reduced TE activity are traded-off against increasing costs of such suppression with equilibrium being a leaky suppressor. That, however, is an equilibrium model and TE suppression systems may not be at equilibrium [149]. Rather they may be regarded as emergency responses in which any limited degree of suppression is better than none (or grossly imperfect suppression). With the possibility of rapid turnover of suppression systems [149], we may expect TE suppressors to be non-optimised. With no filter expected to be foolproof, and TEs under selection to avoid whatever filters are in place, we expect the evolution of multiple leaky pathways. These mechanisms can be broad, such as the HUSH complex (targets potentially mutagenic TEs), or more specific, like the ZNF-KRAB (targets specific TEs) system. Importantly, this leakiness allows TE colonization even in the absence of predispositional escape mechanisms, although naturally we expect that TEs with such predispositions to be more likely to colonize.

We should also highlight that our hypothesis, demonstrating how HERVH has evaded multiple host-encoded filtering mechanisms, aligns with the 'transposon addiction hypothesis' proposed by Cedric Feschotte [150]. Following its invasion, HERVH likely persisted due to genetic drift. However, a subset of genomic copies within the LTR7-HERVH subfamily acquired crucial functions that the host became dependent on. This dependency prevented the silencing of the LTR7-HERVH subfamily, and may explain why HERVH transcripts are highly expressed during early development.

In the case of HERVH this problem is especially notable as the transcripts are both strikingly abundant in some cell types and would in the recent evolutionary past have been prime candidates for suppression.

The above close consideration suggests that predisposing features of HERVH that avoid filters may well be key. In some cases, filter avoidance and novelty generation are intimately coupled. It is notable that both KLF4 enables novel pluripotent functionality and excludes suppressors. Similarly, HERVH is peculiar in having a strong splice site that enables generation of chimeric multi-exon transcripts. By all accounts these should pass the filters to enable, for example, nuclear export of multi-exon transcripts.

In other regards HERVH appears to have evolved mechanisms to escape classic suppression systems. It is noteworthy that many suppression mechanisms that originally developed in response to TE colonization have now been integrated into general cellular quality control processes and now also fulfil additional tasks [11]. For instance, key regulatory innovations in eukaryotic genomes, including introns, splicing, and nuclear export, are widely believed to have emerged, at least in part, as adaptations to TE invasion [151–153]. Other examples include the TP53 pathway, which initially functioned to suppress TE-induced genomic instability via the DNA damage response [154] but has since become a master regulator of the cell cycle, orchestrating apoptosis and senescence in response to DNA damage. Similarly, some KRAB-ZFPs, originally dedicated to TE repression, have been repurposed to regulate host genes during embryonic development. The HUSH complex, once solely responsible for silencing newly integrated transposons [155], now also functions in filtering out noisy transcripts [11, 109] and plays a broader role in regulating immune response genes, neuronal function, and maintaining epigenetic stability during early development.

As regards the effects of m6A, it appears to have taken an anti-unwanted transcript filter system and turned it on its head. Similarly, that HERVH is unusual in avoiding canonical TP53 bindings sites is consistent with a model that you cannot generate novelty unless you can avoid suppression, although in this instance novelty and suppression are not so obviously coupled. Note that while we regard these features as predispositions, they would only be that if these features were ancestral. Otherwise, they would be regarded as evolved features. For our model we consider that predispositions to evasion of suppression is not necessary but will tilt the balance towards domestication. Other suppression evasion mechanisms may come via mutation after the initial active phase and may indeed be favoured as adaptive means to avoid suppression. We often cannot be sure which are evolved and which ancestral but as some level of gene expression is necessary for initial co-option, it is likely that some are ancestral, similar survival bias potentially explaining why nuclear and cytoplasmic viruses have different nucleotide contents [156]. Note that even if ancestral it is not necessary to evoke the hypothesis that these ancestral states are the product of selection favouring expression in the new host, but rather that, present for whatever reason, they further such expression. It remains an open question as to whether, when TE’s move between organisms, selection acting in one host predisposes to successful colonization of a subsequent host.

One should also note that many non-functional HERV-derived sequences in the genome likely evade defense mechanisms. Over time, these sequences undergo stochastic loss and divergence, eventually losing their resemblance to HERVs, posing no threat, and no longer being recognized as ‘non-self.’ However, some HERVs, despite being fixed for millions of years, remain detectable by host defense machineries under pathological conditions.

This also applies to HERVs with co-opted functions. Notably, tolerance to HERVH-derived transcripts is lost during differentiation, suggesting that even co-opted functions, typically recognized as ‘self’, may lose host tolerance under certain conditions. This implies that the balance between immune tolerance and self-defense activation is shaped by factors such as developmental stage, cellular environment, or pathological conditions. Notably, pseudogenes and intronic sequences, despite lacking function, are unequivocally recognized as ‘self’.

Understanding these problems is relevant, we suggest, not simply in an evolutionary context but also in the context of transgene design, and indeed especially relevant in the context of gene therapies that seek to incorporate genes stably in to host DNA (less so for RNA vaccines as these need have no transcriptional or nuclear export components). Indeed, non-expression of some transgenes is common, in part because they are thought to be captured by unwanted transcript filters [11]. Are these being treated like a TE with a potential gain of function but falling foul of the transcript filters?

The same insights might also have relevance for cancer pathology. Cancer cells are often thought to resemble in many regards pluripotent cells [70, 157]. Key transcription factors that govern early embryonic fate are also active in certain tumor cells [158]. Deregulated chromatin accessibility may be a common factor linking cancer to early embryogenesis. As HERVH is active in such cells and appears to have anti-unwanted transcript features it may well be a candidate for gain of function not only in evolutionary time but in tumour-time (for want of a better expression). While much TE expression in cancers may reflect nothing more than unwanted transcripts enabled by disturbed epigenetic regulation, the chimeric HERVH-CALB1 transcript detected in cancer [159], has also been observed in human embryonic stem cells and in the epiblast of preimplantation embryos [52, 160], suggesting that it may represent an exaptation event in normal physiology and also functional in cancer-time. Pathologically activated ERVs can be repurposed as alternative promoters that drive oncogene expression [161–163], sometimes referred to as onco-exaptation. Abnormally activated ERVs may allow cancer cells to exploit and repurpose developmental pathways promoting malignancy [164]. For example, in colorectal cancer, the loss of the BAF chromatin remodeler subunit ARID1A, a tumor suppressor, leads to the derepression of a specific set of HERVH loci, which in turn modulates BRD4-dependent transcription [165]. These onco-exaptation events are often linked to poorer patient outcomes.

In contrast to typical onco-exaptation, a role favourable to the host was observed for the protein product (*Rec*) of the evolutionarily younger HERVK, which counteracts the progression of the cancer to an invasive stage [166]. Additionally, in lung squamous cell carcinoma (LUSC), the production of chimeric transcripts driven by HERVH and a neighboring gene from the calbindin 1 (CALB1) locus correlates with improved patient survival [159].

Understanding the cancer co-options – both harmful and beneficial – may provide an accessible system to more generally understand how ERV related transcripts both generate novelty and escape unwanted transcript suppression systems. This however comes with an important caveat, namely that, as many cancers are characterised by mutations in the unwanted transcript filters [167] (indicative of the importance of such filters), gain-of-function in cancers need not always be a good model of gain-of-function over evolutionary time when these filters would be operative.

## Supplementary Information

Supplementary Material 1: Table S1. Upregulated HERVH family members upregulated in KO TRIM28 hESCs (data source from [132]). Note that the top expressed ESRG locus is not affected.

Supplementary Material 2: Table S2. Frequency of near-perfect TP53 sites (Two 10-mer (10 nucleotides (nt)) half-sites, linked by a short spacer region of 0–13 nt) in selected ERVs in the human genome (data source from [132]).

## Data Availability

No datasets were generated or analysed during the current study.
